# Performance Comparison of Ultrasound-Based Methods to Assess Aortic Diameter and Stiffness in Normal and Aneurysmal Mice

**DOI:** 10.1371/journal.pone.0129007

**Published:** 2015-05-29

**Authors:** Bram Trachet, Rodrigo A. Fraga-Silva, Francisco J. Londono, Abigaïl Swillens, Nikolaos Stergiopulos, Patrick Segers

**Affiliations:** 1 IBiTech-bioMMeda, Ghent University-IMinds Medical IT, Ghent, Belgium; 2 Institute of Bioengineering, Ecole Polytechnique Federale de Lausanne, Lausanne, Switzerland; Medical University Innsbruck, AUSTRIA

## Abstract

**Objective:**

Several ultrasound-based methods are currently used to assess aortic diameter, circumferential strain and stiffness in mice, but none of them is flawless and a gold standard is lacking. We aimed to assess the validity and sensitivity of these methods in control animals and animals developing dissecting abdominal aortic aneurysm.

**Methods and Results:**

We first compared systolic and diastolic diameters as well as local circumferential strains obtained in 47 Angiotensin II-infused ApoE ^-/-^ mice with three different techniques (BMode, short axis MMode, long axis MMode), at two different abdominal aortic locations (supraceliac and paravisceral), and at three different time points of abdominal aneurysm formation (baseline, 14 days and 28 days). We found that short axis BMode was preferred to assess diameters, but should be avoided for strains. Short axis MMode gave good results for diameters but high standard deviations for strains. Long axis MMode should be avoided for diameters, and was comparable to short axis MMode for strains. We then compared pulse wave velocity measurements using global, ultrasound-based transit time or regional, pressure-based transit time in 10 control and 20 angiotensin II-infused, anti-TGF-Beta injected C57BL/6 mice. Both transit-time methods poorly correlated and were not able to detect a significant difference in PWV between controls and aneurysms. However, a combination of invasive pressure and MMode diameter, based on radio-frequency data, detected a highly significant difference in local aortic stiffness between controls and aneurysms, with low standard deviation.

**Conclusions:**

In small animal ultrasound the short axis view is preferred over the long axis view to measure aortic diameters, local methods are preferred over transit-time methods to measure aortic stiffness, invasive pressure-diameter data are preferred over non-invasive strains to measure local aortic stiffness, and the use of radiofrequency data improves the accuracy of diameter, strain as well as stiffness measurements.

## Introduction

In recent years, mice have become the animal model of choice to study cardiovascular disease in general[1], and abdominal aortic aneurysm (AAA) in particular[2, 3]. The murine cardiovascular system bears a strong resemblance to the human one [4], and the possibility to induce an aneurysm in otherwise healthy mice allows for testing of possible pharmacological treatments in longitudinal studies [5–8]. However, these advantages come at a cost: due to the small size and fast heart rate of mice, in vivo imaging of their cardiovascular system requires a much higher spatial and temporal resolution than what is the case for humans. Dedicated small animal imaging technologies such as contrast-enhanced micro-CT[9, 10], micro-MRI[11–13] and high-frequency ultrasound[14] have been developed to address this need. High-frequency ultrasound is by far the least expensive and the most versatile of these tools. It can be used to assess, among others, aortic morphology and dimensions (B-Mode imaging), the change in aortic diameter throughout the cardiac cycle (M-Mode imaging) and blood velocity (Pulsed Doppler imaging) at virtually all locations in the arterial tree.

Within this paper, we focus on the use of high-frequency ultrasound to quantify anatomical (i.e. aortic diameter) and functional (i.e. circumferential strain, aortic distensibility, aortic pulse wave velocity) properties of normal and aneurysmal arteries in mice. Short axis BMode (tracking either the circumferential perimeter [15–18] or the linear diameter [19–22]) and long axis MMode (tracking the linear diameter with superior time resolution [15, 23–25]) are often used to quantify and track AAA dimensions over time as a measure for AAA severity. Aortic diameter as measured by high-frequency ultrasound has been reported as reliable [21, 26, 27] with small variability [20]. Ultrasound is, however, a highly operator-dependent technique that requires a sound anatomical knowledge of the imaged structures by the operator. Moreover the diameter measurements obtained with ultrasound can vary considerably depending on the used application (BMode, MMode), imaging angle (short axis, long axis) and measurement method (circumferential perimeter, linear diameter).

Aortic stiffness has been assessed in vivo in mice by local methods such as circumferential cyclic Green-Lagrange strain [15, 28, 29], pulse wave imaging [30–32] and diameter-velocity loops [33, 34]. Alternatively, global or regional methods can be used to assess the aortic pulse wave velocity (PWV), a surrogate measure to assess aortic stiffness over a larger part of the aorta. PWV is, however, more challenging to measure in mice than in humans. The transit time between two arterial sites can be measured invasively with one double-sensor [35] or two single-sensor pressure catheters[36], or noninvasively via tonometry [37], magnetic resonance imaging (MRI) [38, 39] or high-frequency ultrasound [33, 34, 40, 41]. The corresponding distance is known for a double-sensor pressure catheter and MRI, but has to be estimated from external body measurements for the other methods. Despite the fact that murine PWV is used as a marker for arterial stiffness in many papers, the distance measurements often suffer from a high measurement error (relative to the low absolute value of the distance measured), while an invasive probe alters the local hemodynamics and MRI has limited temporal resolution. Therefore a gold standard for measuring PWV in mice is lacking.

In previous (unpublished) research, we encountered a large variability when quantifying both aortic diameters and aortic stiffness in angiotensin II-infused aneurysmal mice. To better understand the observed differences and the underlying anatomy, we decided to set up a novel study to visualize the AAAs ex vivo with X-ray synchrotron radiation. Combining an isotropic resolution of 6.5 micron with soft tissue contrast, these images demonstrated that aneurysmal mice suffer from an apparent luminal dilatation due to a tear in the tunica media near the celiac artery [42]. Additionally, an intramural hematoma that originates from ruptured suprarenal aortic side branches dissects the adventitia from the media. This increased insight on how to interpret the underlying anatomy of murine dissecting AAA also shed a new light on the in vivo ultrasound images of these vascular structures, as it allowed us to provide an explanation for the counter-directional vortex that had been observed with Color Doppler [12, 22, 43] and the so-called ‘dissecting flap’ that had been observed on BMode images [15, 18, 44].

In the current paper we demonstrate how these recent observations affect the quantification of the AAA diameter as it has been assessed with ultrasound so far. We point out the misinterpretations that have been made in the past, and compare values obtained with BMode and MMode (in short and long axis views) in a large dataset of ApoE^-/-^ mice. Second, we demonstrate how different methods to assess circumferential strain, aortic pulse wave velocity and aortic distensibility are affected by the dissecting AAA geometry. We compare transit time PWV values obtained from non-invasive pulsed Doppler ultrasound with those from an invasive double-sensor pressure catheter and we propose a novel in vivo method to assess aortic stiffness, combining a pressure catheter with radio frequency (RF) M-Mode ultrasound. We point out pitfalls that should be avoided, and provide guidelines for the in vivo assessment of aortic diameters and stiffness with high-frequency ultrasound in mice, with or without dissecting AAA.

## Methods

### Mice

All the procedures were approved by the Ethical Committee of Canton Vaud, Switzerland (EC 2623.0 for the diameter study, EC 2647.1 for the stiffness study) and performed according to the guidelines from Directive 2010/63/EU of the European Parliament on the protection of animals used for scientific purposes. All surgery and measurements were performed under 1.5% isoflurane anesthesia, while animals were on heating pads and eyes were protected by an ocular gel. To reduce stress after the surgery of osmotic mini-pump implantation animals received an analgesic (buprenorphine at 0.08 mg/kg subcutaneous route), and cage enrichment was provided. In both studies, general behaviour (posture, bright eyes, grooming, presence of a hunched back, piloerection) was checked every day and weight was measured 2 times a week. Mice were monitored 30 minutes after ultrasound measurements and 1 hour after osmotic pump implantation. Humane endpoints were defined in case of unexpected or major change in the behaviour (quantified using a scoring system), weight loss >15% between two adjacent body weighing sessions, infection in the site of osmotic mini-pump implantation, or abnormal changes in the site of infusion (during subcutaneous administration). In the first study, 2 animals were euthanized before the end of the study because humane endpoints had been reached. In the second study, 8 animals were sacrificed by exsanguination (without anesthesia recovery) after the invasive blood pressure measurements. This avoided unnecessary pain or stress that would have resulted from reduced limb functionality after the invasive pressure probe insertion through the femoral artery.

#### Study 1

For the diameter study 47 male ApoE^-/-^ mice on a C57Bl/6J background were purchased at the age of 20 weeks from Janvier (Saint Berthevin, France). All animals were scanned with ultrasound at baseline (day 0). The animals were subsequently implanted a 200 μl osmotic pump (model Alzet 2004; Durect Corp, Cupertino, CA), filled with Angiotensin II or a combination of Angiotensin II with either Angiotensin-(1–7) or (D-Ala⁷)-Angiotensin-(1–7) (all of them purchased at Bachem AG, Bubendorf, Switzerland) diluted in sterile saline 0.9%. Animals were subdivided into 3 groups to study the effect of the aforementioned peptides on aneurysm incidence and phenotype Nevertheless, all animals developed similar aneurysm phenotypes with no significant differences between groups. As such, data were pooled for the purpose of this methodological study. Eleven animals died from aneurysm rupture in the first 14 days, and 1 additional animal died in the last 14 days. Necropsy was performed to confirm hemoabdomen or hemothorax as the cause of death. A rupture rate of 20% within the first weeks of angiotensin II infusion is in agreement with previously published values [22, 45, 46]. Hence 34 animals were scanned with ultrasound 14 days after pump implantation, and 33 animals were scanned a third time after 28 days.

#### Study 2

For the aortic stiffness study 30 male C57Bl\6J mice were purchased at the age of 12 weeks from Janvier (Saint Berthevin, France). Ten animals served as controls and did not get a pump implanted. In nine control animals aortic transit time PWV and local aortic distensibility were assessed with both ultrasound and invasive pressure. One control could only be imaged with high-frequency ultrasound due to a technical problem preventing pressure probe insertion. In the remaining 20 animals, pumps were implanted similar to the diameter study, and systemic neutralization of TGF-β in the PWV study was achieved by intraperitoneal injections of mouse anti—human TGF-β (2G7 clone[47], 20 mg/kg, three times a week). Six animals died of aneurysm rupture, confirmed by the presence of extravasated blood in the abdominal region (hemoabdomen) or the thoracal cavity (hemothorax), before they could be imaged. A rupture rate of 30% in these animals is in line with literature, since anti-TGF-β injected animals are known to be more susceptible to transmural rupture than the more commonly used angiotensin II-infused, ApoE-^/-^ mice [12, 47]. One animal died during pressure catheter insertion, two animals were sacrificed at day 4 (without AAA presence) and not included in the study, and three animals did not respond to the treatment and did not develop any AAA. Since the remaining 8 AAA mice had to be sacrificed after pressure catheter insertion, these animals could not be followed over time. Aneurysm transit time PWV and local stiffness were therefore assessed in different animals at various stages of aneurysm development (confirmed with high-frequency ultrasound) in order to obtain a wide range of material properties. Two animals were tested (and sacrificed) at day 7, two at day 10, two at day 15 and two at day 22.

### Ultrasound imaging

Ultrasound imaging was performed with a high-frequency ultrasound device (Vevo 2100, VisualSonics, Toronto, Canada) using a linear array probe (MS 550D, frequency 22–55 MHz). During the procedure animals were anesthetized by inhalation of 1.5% isoflurane, and fixed on the imaging table in dorsal position.

**Fig 1:**
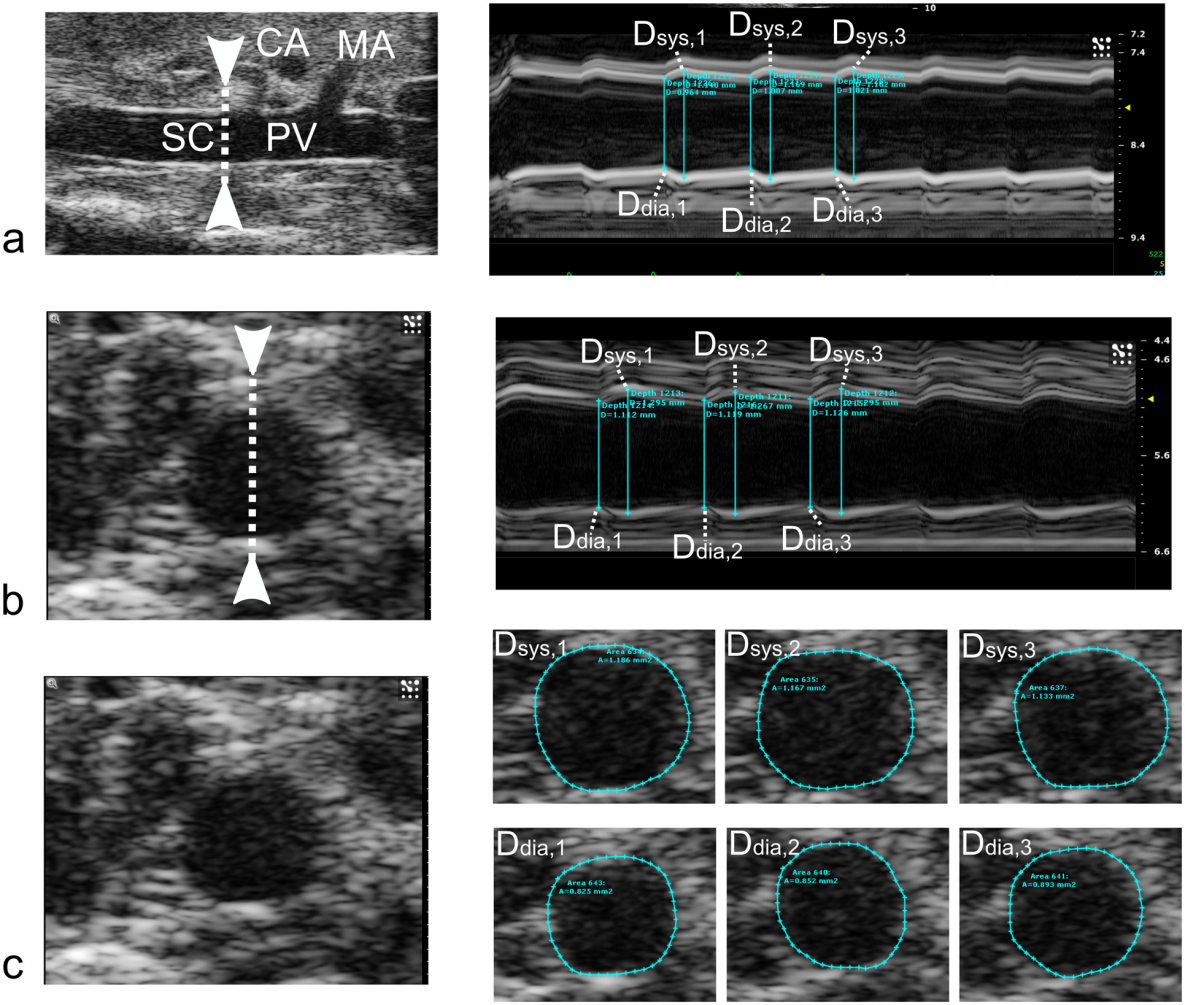
Diameter assessment in three control aortas using (a) Long Axis MMode, (b) Short Axis MMode, and (c) Short Axis BMode. For each case, the images on the right show either diameters (MMode) or circumferences (BMode) at three timepoints during systole and three timepoints during diastole. Tracing was performed using the built-in Vevo 2100 software. For the MMode cases (a and b), the arrows in the left images indicate the placement of the MMode measurement line. Diameter values for the BMode view were derived from the perimeter assuming a circular shape. In all cases, the median diameter from each set of three measurements was used. Branches are labeled in the Long Axis MMode (CA: Coeliac Artery, MA: Mesenteric artery. SC: Supraceliac. PV: Paravisceral), but cannot be visualized in the same field of the view during Short Axis measurements."

**Fig 2:**
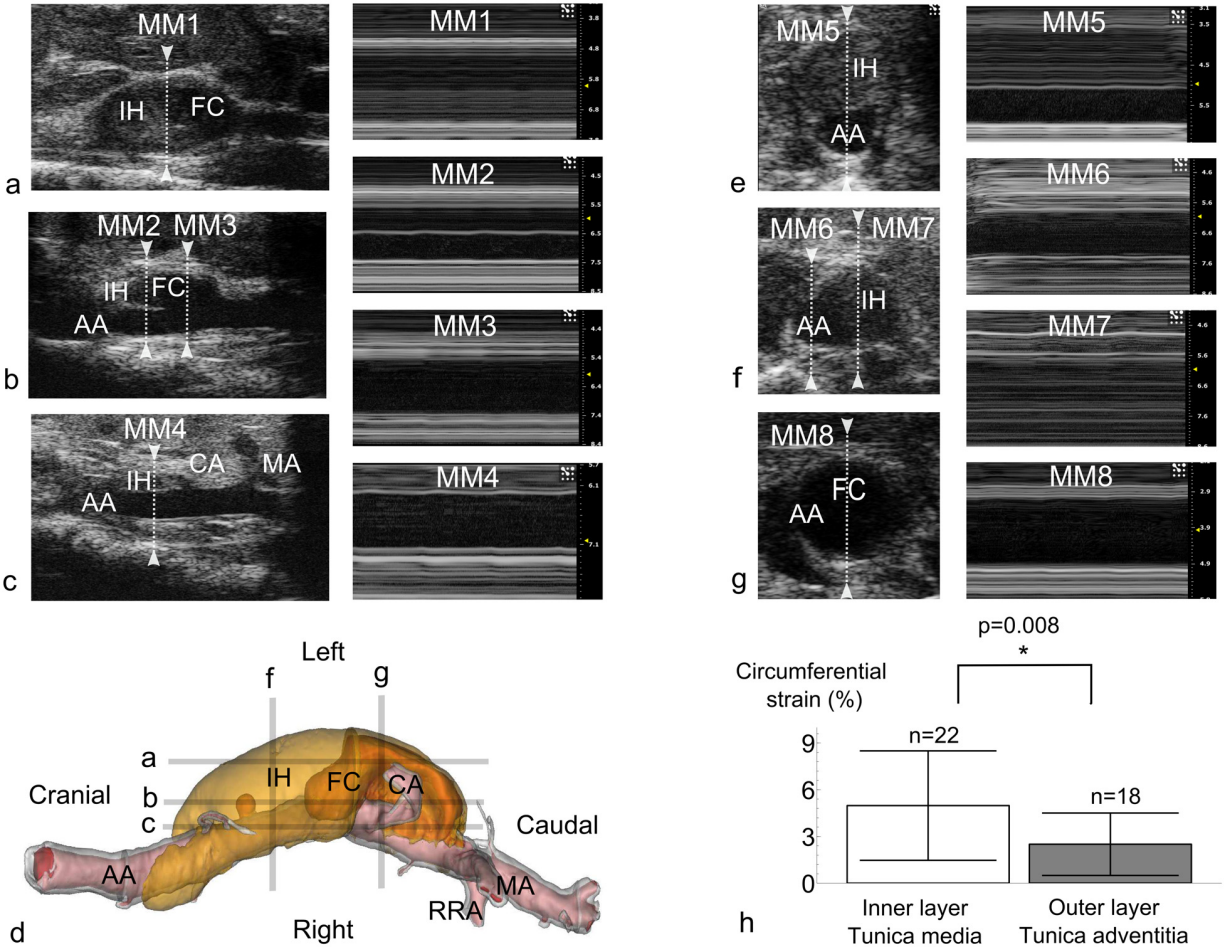
Diameter assessment in dissecting AAAs. **a**. Long axis view taken at the level of the intramural hematoma and false channel, where the abdominal aorta is not visible. **b**. Long axis view taken at the level of the tear in the tunica media, showing a dissecting flap. The image in MM2 shows how the inner layer (tunica media) is more distensible than the dissected outer layer (tunica adventitia), while the tunica media is not visible when measuring at MM3. **c**. Long axis view taken at the level of the aortic lumen, the false channel is not visible, and the intramural hematoma seems much smaller than in reality. **d**. 3D visualization of dissecting AAA based on PCXTM scans. Image taken from [42] for visualization purposes. The ultrasound images shown in panels a-c (LA MMode) and d-f (SA MMode) do not correspond to the actual 3D geometry shown on the top panels, but were taken at locations that were similar to the indicated lines. IH: Intramural Hematoma. FC: False Channel. CA: Celiac Artery. MA: Mesenteric Artery. RRA: Right Renal Artery. AA: Abdominal Aorta. **e**. Short axis view showing an intramural hematoma that is located on the anterior side of the aortic lumen. **f**. Short axis view showing an intramural hematoma that is located on the left side of the abdominal aorta. **g**. Short axis view taken at the level of the false channel. **h**. Short axis MMode circumferential strain in matched pairs (n = 19) where both a dissected adventitia and tunica media could be measured simultaneously (cfr. MM2). The inner, medial layer is more distensible than the outer, adventitial layer. Since the pulse wave travels over the innermost layer, this will influence the PWV value obtained with transit time methods.

In the diameter study (study 1), short axis BMode images of the abdominal aorta were obtained at the supraceliac region just cranial to the bifurcation of the celiac artery, and at the paravisceral region just cranial to the trifurcation of the mesenteric and right renal arteries. MMode images were obtained in short axis at the exact same locations. Afterwards, the probe was turned 90 degrees and MMode images were obtained at both the supraceliac and the paravisceral regions in the long axis view (Fig 1, for controls, and Fig 2, for AAAs).

**Fig 3:**
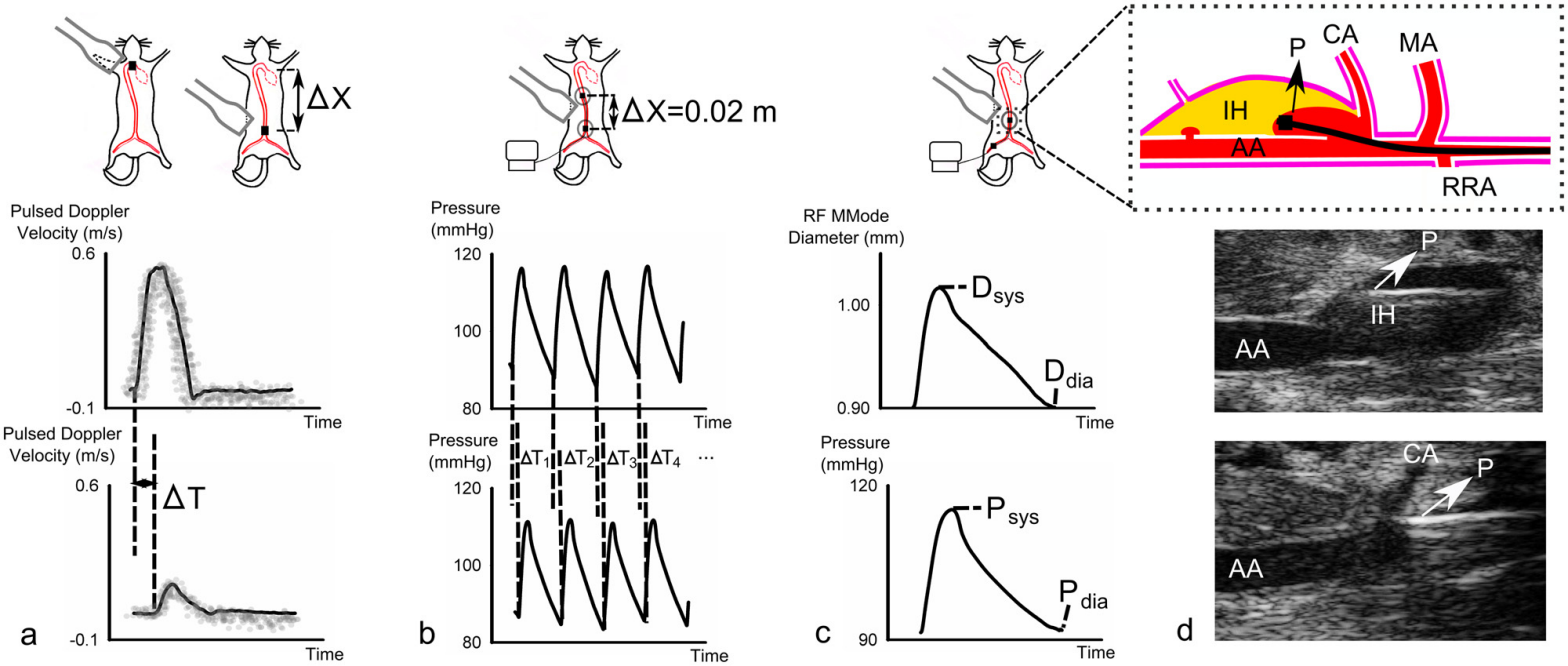
PWV assessment in controls and dissecting AAAs. **a**. Global, ultrasound-based transit time PWV (Eq (3)). Descending and distal abdominal pulsed Doppler waveforms were obtained sequentially, the foot-to-foot time lag is obtained from averaged waveforms. The distance between both measurement locations is measured externally with tape. **b**. Regional, pressure-based transit time PWV (Eq (4)). Pressure waveforms are obtained simultaneously, and the foot-to-foot time lag from beat-to-beat waveforms. The distance between both pressure sensors is fixed on 0.02m. **c**. Local PWV assessed from aortic distensibility (Eqs (5) and (6)). Invasive pressure waveforms and non-invasive, raw-frequency MMode diameter waveforms are obtained simultaneously. No distance measurement is needed. **d**. Top: schematic representation depicting how in some cases the inserted pressure probe entered into the false channel at the level of the tear in the tunica media. IH: Intramural Hematoma. FC: False Channel. AA: Abdominal Aorta. P: Pressure probe. Since further insertion of the probe was not possible, no transit time PWV could be obtained in these cases. Middle: In vivo long axis BMode image showing how the pressure probe gets stuck within the intramural hematoma of a dissecting AAA. Bottom: In vivo long axis BMode image of the pressure probe inserted inside a normal aorta without dissecting AAA.

In the PWV study (study 2), aortic material properties were assessed in three different ways. First, global PWV was measured noninvasively over the entire aorta. Pulsed Doppler images were obtained at the ascending aorta (just cranial to the aortic valve) and the abdominal aorta (just cranial to the iliac bifurcation). Subsequently the distance between both locations was approximated by an external body tape measurement (Fig 3a). Then regional PWV was assessed invasively at the abdominal aorta. A 1.2 F Scisense catheter (Transonic, Maastricht, The Netherlands) with two pressure sensors spaced 2 cm apart was inserted via an incision in the femoral artery. The catheter was guided into the correct position using simultaneous BMode ultrasound, and allowed to settle for 10 minutes. Simultaneous pressure waveforms were first recorded with the tip of the catheter (i.e. the first pressure sensor) at the descending aorta, measuring regional thoracic PWV, and afterwards with the tip of the catheter at the supraceliac region, measuring regional abdominal PWV (Fig 3b). Finally, local aortic distensibility was assessed invasively at the supraceliac abdominal aorta. The pressure catheter was positioned using long axis BMode imaging, and the diameter variation over time was recorded with RF long axis MMode while the local pressure variation was recorded simultaneously. During each pressure-diameter measurement, a synchronization signal was sent out from the data acquisition software to the Vevo 2100 machine (Fig 3c).

### Data processing

In the diameter study, BMode ultrasound data were processed within the Vevo 2100 analysis software. For each BMode measurement three different cardiac heart beats were processed, and for each cardiac cycle the diameter perimeter was traced manually at peak systole and peak diastole, using the built-in Vevo 2100 diameter tracing tool (Fig 1c). Diameter values were calculated from perimeter values assuming a circular circumference. For three different cardiac cycles of each short axis MMode (Fig 1b) and long axis MMode (Fig 1a), the maximum diameter was traced manually at peak systole and the minimum diameter at end diastole, using the built-in Vevo 2100 linear distance measurement tool. All values were then exported into Matlab (The Mathworks, Natick, MA), and for each measurement location the median value over three cardiac cycles was selected. Unless mentioned otherwise, all presented diameters at all time points represent the outer diameter, i.e. the outline of the tunica adventitia (Fig 2). For all methods, systolic and diastolic diameters were combined to quantify the circumferential cyclic Green-Lagrange strain [28, 48], under the assumption of uniform strain around the vessel (Eq (1), Fig 1). In those cases where the original inner and the dissected outer layer where clearly discernible on the same image (e.g. Fig 2b, MM2), circumferential strains were assessed for both layers (Fig 2h). Different methods to assess aortic diameter and strain were subsequently compared with bar plots, correlation and Bland-Altman plots. A two-tailed Student’s t-test was performed to compare different groups, with p<0.05 considered significant. Pearson’s correlation coefficient was calculated between different methods, as well as the coefficient of variation (CV, Eq (2)). The latter was calculated as the standard deviation of the differences between two measurements (D_1_ and D_2_), normalized by their mean value.

$$Circ.strain=\frac{1}{2}\left[{\left(\frac{D_{sys}}{D_{dia}}\right)}^{2}-1\right]*100\%$$ (1)

$$CV=\frac{\sqrt{\frac{1}{n-1}\sum_{i=1}^{n}{\left(D_{2}(i)-D_{1}(i)\right)}^{2}}}{\frac{1}{n}\sum_{i=1}^{n}\frac{\left(D_{2}(i)+D_{1}(i)\right)}{2}}*100\%$$ (2)

$$PWV_{global,ultrasound}=\frac{\Delta x}{\Delta T}$$ (3)

$$PWV_{regional,pressure}=\frac{0.02}{\frac{1}{n}\sum_{i=1}^{n}\Delta T_{i}}$$ (4)

$$Distensibility=\frac{D_{sys}^{2}-D_{dia}^{2}}{(P_{sys}-P_{dia})*D_{dia}^{2}}$$ (5)

$$PWV_{local,pressure-diameter}=\frac{1}{\sqrt{\rho \cdot Distensibility}}$$ (6)

Ultrasound Pulsed Doppler waveforms were traced within a custom-made environment platform in Matlab. The average of three different waveforms was determined and the time difference between the R-top of the ECG-signal and the foot of the flow velocity wave was calculated for both the ascending and abdominal velocity measurement. Global, ultrasound-based pulse wave velocity (Eq (3), Fig 3a) was subsequently calculated as the ratio of the tape-measured distance between both locations (Δx) over the foot-to-foot transit time between averaged waveforms at both locations (ΔT). Invasive pressure measurements were processed in Labchart (AD Instruments, Dunedin, New Zealand). Foot-to-foot transit times were determined for every cardiac cycle separately, and regional, pressure-based PWV was calculated for both the thoracic and the abdominal aorta as the ratio of distance (2 cm between both catheters) over the transit time that was averaged over 20 heart beats (Eq (4), Fig 3b). Finally a custom-written, Matlab-based application was used to obtain the aortic distensibility from the RF MMode data. For each measurement, 3 cardiac cycles were selected and plotted as a reference for semi-automatic wall segmentation. The algorithm tracks the arterial distension using a modified autocorrelation estimator, which accurately estimates the tissue velocities by compensating for a potential downshift of the received centre frequency due to attenuation [49]. For both anterior and posterior walls, a tracking line was calculated based on a user-defined starting position. As will be shown in the results section, the tracked aortic wall movement yields accurate in vivo diameter waveforms, even for very stiff vessels such as the abdominal aneurysms. The averaged diameter distension waveform was subsequently combined with the pressure waveform (averaged over 20 cycles) that was measured simultaneously, and local aortic area distensibility (Eq (5), Fig 3c) and PWV (Eq (6)) were calculated.

## Results

### Diameter assessment with high-frequency ultrasound

**Fig 4:**
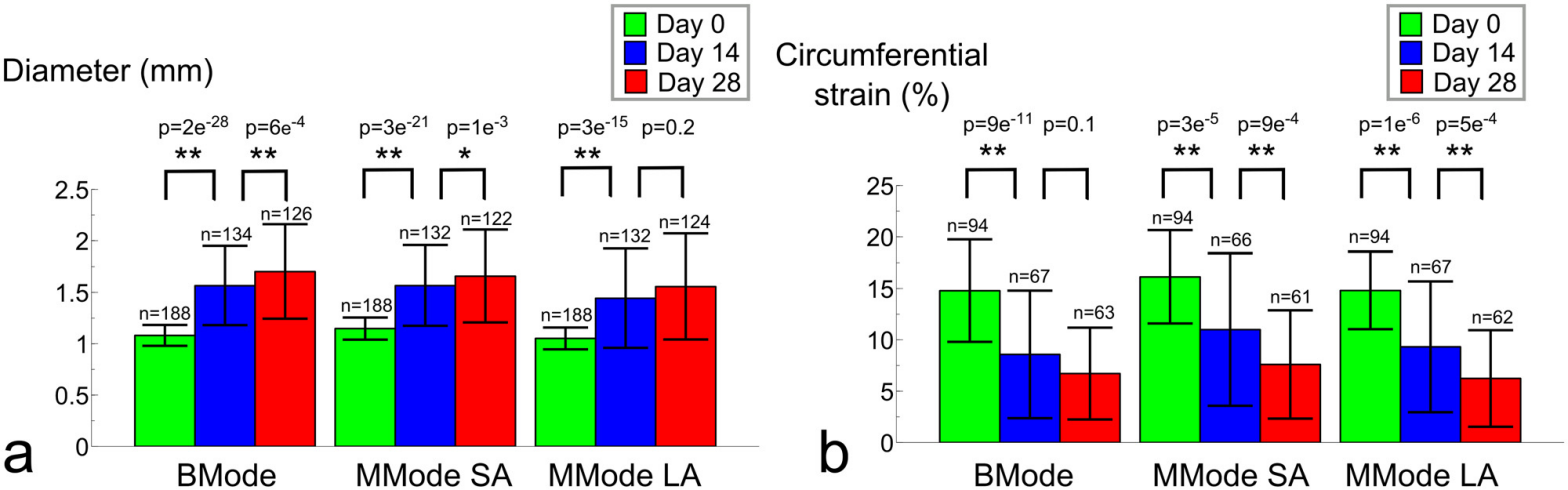
Increase of aortic diameters and decrease of circumferential strains over time. **a**. Aortic diameters are shown for 3 different methods, measured at paravisceral and supra-celiac regions and showing systolic as well as diastolic values. All methods show low standard deviations at baseline but not at later time points. All methods find a highly signficant difference (p<0.001, indicated by **) between diameters at baseline and day 14. Short axis BMode and MMode find a highly significant difference between diameters at days 14 and 28, but long axis MMode does not. **b**. Circumferential strains are shown for 3 different methods, measured at paravisceral and supra-celiac regions. All methods show high standard deviations at all time points. All methods find a highly significant difference between diameters at baseline and day 14. Short axis and long axis MMode find a highly significant difference between strains at days 14 and 28, but BMode does not.

**Fig 5:**
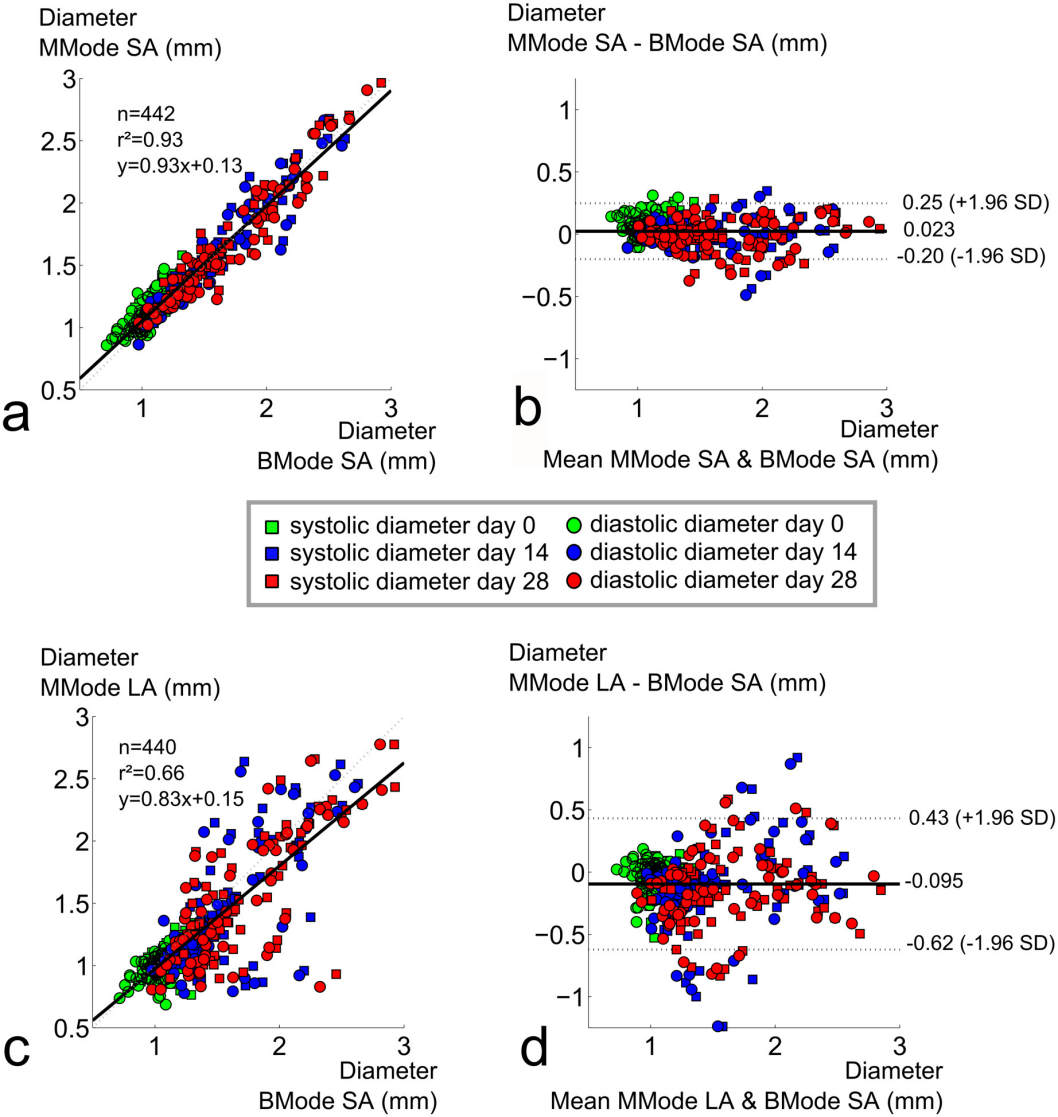
Comparison of diameters assessed with short axis BMode to long axis or short axis MMode. **a**. High correlation between short axis MMode and BMode diameters. **b**. Bland-Altman plot shows low dispersion between short axis MMode and BMode diameters, no bias. **a**. Poor correlation between long axis MMode and BMode diameters. **b**. Bland-Altman plot shows high dispersion between long axis MMode and BMode diameters, no bias, larger dispersion for later time points.

For all methods in which the aortic diameter was measured with high frequency ultrasound, diameters increased as aneurysms developed (Table 1, Fig 4a). Coefficients of variation were small at baseline for all methods (BMode:9%; SA MMode:9%; LA MMode:10%), but increased with aneurysm presence at days 14 (BMode:25%; SA MMode:25%; LA MMode:33%) and 28 (BMode:27%; SA MMode:27%; LA MMode:33%) (Fig 4a). All methods detected a highly significant difference in aortic diameter between baseline and day 14 (p<0.001). BMode imaging was able to detect a highly significant difference in aortic diameter between days 14 and 28 as well. This difference was still significant for short axis MMode, but both end-stage timepoints could not be differentiated with long axis MMode. Moreover, a good overall correlation was found between short axis BMode and short axis MMode diameters (r² = 0.93, Fig 5a) and the Bland-Altman plot showed low dispersion between both methods (CV = 8%, Fig 5b). When comparing both short axis methods for different time points separately, the agreement was less prominent at baseline (n = 188, r² = 0.63), but mainly driven by the good correspondence between aneurysmal diameter values measured at days 14 (n = 132, r² = 0.90) and 28 (n = 122, r² = 0.92). There was no systematic bias nor proportional error, and the variation did not depend on the magnitude of the measurements.

**Table 1:** Diameters and circumferential strains obtained with three techniques at three different time points and two different aortic locations.

		SampleSize SC/PV	D_sys_ SC (mm)	D_dia_ SC (mm)	D_sys_ PV (mm)	D_dia_ PV (mm)	Circ. Strain SC (%)	Circ. Strain PV (%)
BMode	D0	47/47	1.19 ± 0.11	1.05 ± 0.10	1.11 ± 0.08	0.98 ± 0.9	15.4 ± 4.6	14.2 ± 5.3
	D14	34/33	1.67 ± 0.40	1.56 ± 0.45	1.57 ± 0.33	1.46 ± 0.35	9.1 ± 7.6	8.1 ± 4.3
	D28	33/30	1.83 ± 0.50	1.73 ± 0.52	1.66 ± 0.38	1.57 ± 0.40	6.9 ± 5.0	6.5 ± 3.9
SA MMode	D0	47/47	1.27 ± 0.10	1.11 ± 0.11	1.18 ± 0.09	1.03 ± 0.09	16.0 ± 4.5	16.2 ± 4.6
	D14	34/32	1.68 ± 0.38	1.55 ± 0.44	1.58 ± 0.35	1.45 ± 0.40	10.9 ± 7.6	11.1 ± 7.4
	D28	32/29	1.77 ± 0.49	1.67 ± 0.42	1.64 ± 0.37	1.54 ± 0.40	7.7 ± 5.8	7.5 ± 4.7
LA MMode	D0	47/47	1.16 ± 0.11	1.02 ± 0.10	1.07 ± 0.09	0.95 ± 0.09	15.3 ± 3.8	14.3 ± 3.7
	D14	34/33	1.56 ± 0.49	1.46 ± 0.53	1.43 ± 0.45	1.32 ± 0.46	9.2 ± 6.4	9.5 ± 6.4
	D28	33/29	1.74 ± 0.55	1.67 ± 0.58	1.43 ± 0.39	1.35 ± 0.40	5.9 ± 5.4	6.6 ± 3.8

SC: Supra-celiac. PV: Paravisceral. SA: Short Axis. LA: Long Axis. Mean values ± standard deviation.

The correlation between short axis BMode and long axis MMode diameter, on the other hand, was much less convincing (r² = 0.66, Fig 5c) and the overall dispersion on the Bland Altman plot much higher (CV = 19%, Fig 5d). Similar results were obtained between short and long axis MMode methods (r² = 0.64, CV = 19%, Fig. not shown). When comparing BMode with long axis MMode for different time points separately, the overall correlation was lowest at baseline (r² = 0.45) but the Bland-Altman plots revealed that the dispersion was also much lower (CV = 9%). At later time points, corresponding to aneurysm presence and thus higher diameter values, the correlation was slightly better (r² = 0.53 at day 14 and r² = 0.59 at day 28). However, the dispersion between both methods was also higher at later time points, which was reflected by a higher coefficient of variation at both days 14 (CV = 22%) and day 28 (CV = 20%).

### Circumferential strain assessment with high-frequency ultrasound

**Fig 6:**
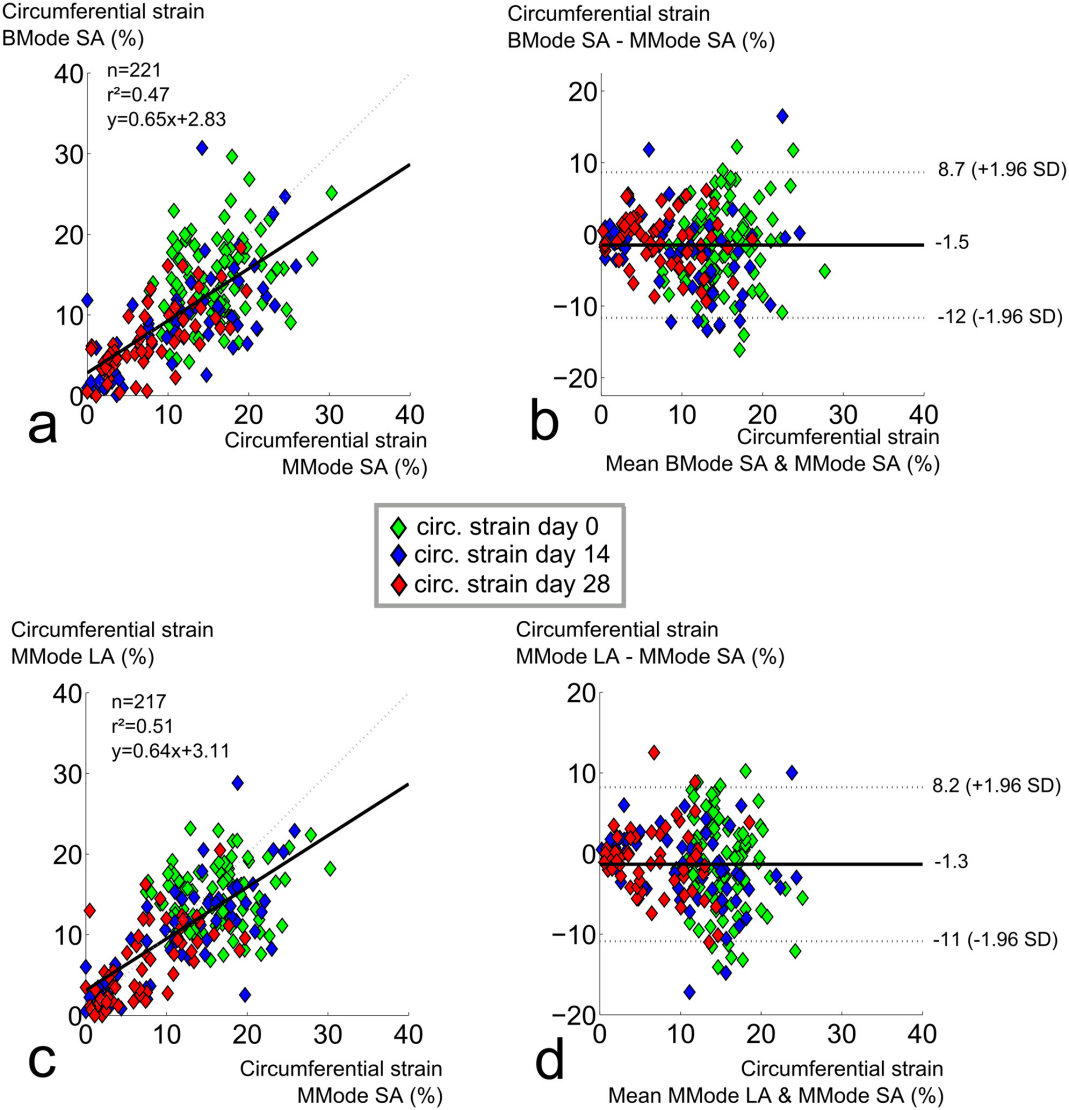
Comparison of circumferential strains assessed with short axis MMode to short axis BMode or long axis MMode. **a**. Poor correlation between BMode and short axis MMode circumferential strains. **b**. Bland-Altman shows high dispersion between short axis BMode and short axis MMode circumferential strains, no bias, larger dispersion for earlier time points. **c**. Poor correlation between long axis and short axis MMode circumferential strains. **d**. Bland-Altman shows high dispersion between long axis MMode and short axis MMode circumferential strains, no bias, larger dispersion for earlier time points.

For all methods to measure circumferential strain with high frequency ultrasound, strains decreased as aneurysms developed (Fig 4b). Standard deviations were high for all methods at all time points (25–35% of the mean value at baseline, 65–75% of the mean value at days 14 and 28). All methods detected a highly significant difference in circumferential strain between baseline and day 14 (p<0.001). Both short and long axis MMode methods detected a highly significant difference in strain between days 14 and 28 as well, but BMode imaging could not differentiate between both end-stage time points. Neither the correlation between short axis MMode and short axis BMode (r² = 0.47, Fig 6a) nor the correlation between short axis MMode and long axis MMode (r² = 0.51, Fig 6c) were very good. This was also reflected in the Bland-Altman plots (CV = 44% and 41%, respectively, Fig 6b and 6d). Correlation was absent at baseline (r² = 0.06 and 0.03 respectively), and modest at best once aneurysm diameters were taken into account (r² = 0.51 and 0.60 at day 14 and r² = 0.52 and 0.44 at day 28). The Bland-Altman plot seemed to indicate a lower variation in circumferential strain for lower strain values. But despite the fact that the absolute value of the 95% confidence interval was lower at later time points, normalization over the lower mean strain still resulted in a higher coefficients of variation at days 14 (CV = 55% and 45%) and 28 (CV = 50% and 60%) than at baseline (CV = 37% and 34%).

### Aortic stiffness assessment with high-frequency ultrasound and invasive pressure

**Fig 7:**
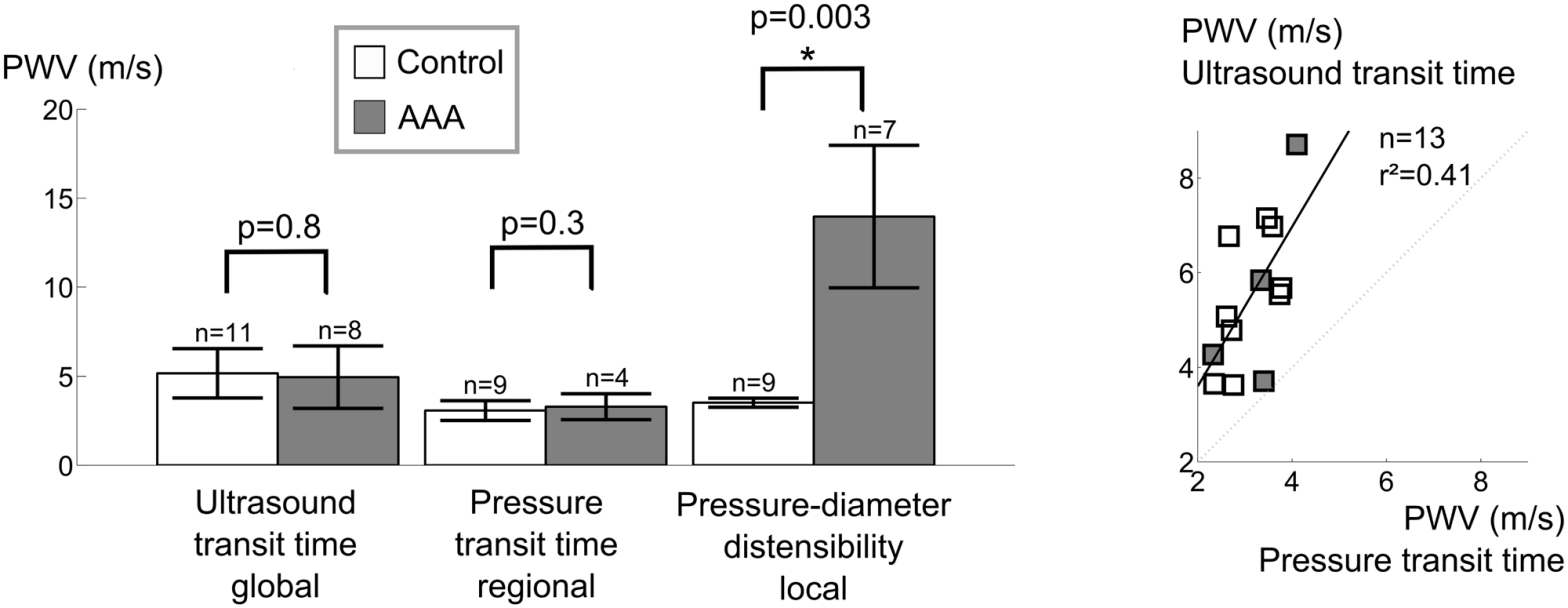
Comparison of different methods to assess aortic PWV in vivo. **a**. Standard deviations in control animals are highest for ultrasound-based transit time and lowest for the pressure-diameter method. Ultrasound nor pressure-based transit time methods can differentiate in PWV between controls and AAAs, while the pressure-diameter method detects a significant difference. **b**. Poor correlation between ultrasound-based and pressure-based transit time methods for PWV assessment. In only 4/8 AAAs a pressure transit time could be obtained (Fig 3d).

For control animals, the coefficient of variation was highest for ultrasound-based transit time (n = 10, PWV = 5.2 ± 1.4 m/s, CV = 27%), relatively high for pressure-based transit time (n = 9, PWV = 3.1 ± 0.6 m/s, CV = 18%) and lowest for local pressure-diameter (n = 9, PWV = 3.5 ± 0.3 m/s, CV = 7%, Fig 7a). For animals with AAA, high coefficients of variation were observed using ultrasound-based transit time (n = 8, PWV = 5.0 ± 1.8 m/s, CV = 35%), pressure-based transit time (n = 4, PWV = 3.3 ± 0.7 m/s, CV = 22%) as well as local pressure-diameter (n = 7, PWV = 14.0 ± 4.0 m/s, CV = 28%). Neither global ultrasound-based nor regional pressure-based transit time methods could differentiate aortic PWV between controls and aneurysmal mice, while a significant difference in local PWV was found using the pressure-diameter technique (p<0.01, Fig 7a). Moreover, we found that ultrasound-based transit time resulted in significantly higher PWV values than pressure-based transit-time PWV. This difference was mainly driven by controls (p<0.001), and was not detectable for animals with AAAs alone. Moreover, both transit time based methods were poorly correlated (r² = 0.41, Fig 7b), and the correlation coefficient decreased further when only controls were taken into account (r² = 0.33).

In 50% of the AAA mice (n = 4/8), the tip of the pressure sensor (which had been inserted into the femoral artery and moved through the aorta in cranial direction) entered into the false channel/intramural hematoma at the level of the celiac artery (Fig 3d). The sensor could therefore not be moved far enough to allow for a measurement from the second sensor and no transit time could be measured in these animals. Circumferential strain measurements show that the inner layer, i.e. the original tunica media delining the abdominal aorta, is significantly more distensible than the outer layer, i.e. the dissected tunica adventitia (Fig 2h).

## Discussion

### Diameter assessment with high-frequency ultrasound

Our data indicate that aortic diameters should be assessed in short axis rather than long axis when using ultrasound in mice. Both short axis methods could differentiate between days 14 and 28, while long axis MMode could not (Fig 4a). Moreover, while both short axis methods show good correlation and low dispersion, long axis MMode did not correlate well with any of the short axis methods, not even at baseline. These observations confirm earlier results reported by Martin-McNulty *et al*., who observed poor correlations (r² = 0.64 for outer diameter and r² = 0.49 for luminal diameter) between long and short axis BMode diameters in angiotensin II-infused mice [19]. Importantly, their diameters were measured along a single line (as is the case for MMode) and not circumferentially, as in our BMode images. Nevertheless, despite the fact that they are often used to quantify AAA size [19–22, 49], we did not include linear BMode diameters in this comparison. If one is to measure diameters along a single line, MMode is the obvious choice as it offers higher temporal and spatial resolution than BMode. Consistent with our findings, Martin-McNulty et al also reported long axis BMode diameters to be lower albeit not significantly different from short axis BMode diameters[19]. We hypothesize that this is related to the nature of long axis measurements. In presence of AAA (at days 14 and 28) the aneurysm bulges out leftward due to the intramural hematoma and false channel formation, and thus the dilated outer wall of the aorta often does not have a circumferentially concentric shape (Fig 2d, 2e and 2f). When assessing the circumferential diameter in short axis BMode, this can be accounted for (Fig 1c). But when assessing aortic diameter along a single line, things are more complicated (Fig 1a and 1b). In short axis, the entire cross-section is visible and it is straightforward to position the measurement axis at the level of maximal diameter (Fig 2e and 2g). In case of asymmetric bulging on the left side of the AAA (Fig 2f), however, the measurement axis cannot be placed at the level of the maximal diameter. This may explain why short axis BMode achieved a slightly higher significance (p = 6.4e^-4^) than short axis MMode (p = 1.3e^-3^) when differentiating between AAAs at days 14 and 28. In long axis on the other hand the field of view has to be shifted in order to find the level of maximal diameter (Fig 2a, 2b, 2c and 2d), which induces a larger error and dispersion in the final diameter values (Fig 4a). Any error in finding the imaging plane corresponding to the maximal diameter, will inevitably result in an underestimation of the obtained diameter value (Fig 4a) This effect is most prominent in presence of asymmetric aneurysms, but occurs even for concentric diameters at baseline. The reason why long axis MMode is still used to assess diameters is that one can easily identify landmarks such as celiac or mesenteric branches and measure within the same field of view (Fig 1a). In short axis, one has to shift the field of view in order to determine the measurement position according to these landmarks (Fig 1b).

### Circumferential strain assessment with high-frequency ultrasound

Our data suggest that circumferential strain should not be assessed with short axis BMode. Unlike both MMode-based methods, BMode could not differentiate between strain values at days 14 and 28 (Fig 4b). One might argue that, rather than demonstrating the superiority of MMode over BMode for the assessment of circumferential strain, our MMode measurements could be false positives that pick up a non-existent stiffening of the aneurysmal aortic wall. Indeed, Haskett *et al*., performed biomechanical testing on angiotensin II-infused aortas and did not find a difference in circumferential strain between 14 and 28 days of angiotensin II infusion [50]. Their data were, however, based on a very limited sample (n = 6), and suffered from a high variability due to large inter-mouse changes in aneurysm morphology. We accounted for this variability by imaging each of the aneurysmal aortas (n = 34 at day 14, n = 31 at day 28) at 2 different aortic locations (SC and PV) and at 2 different imaging angles (SA and LA). It is also important to keep in mind that the limited temporal resolution of BMode does not allow to detect the exact moment of peak systole and (to a lesser extent) diastole with sufficient accuracy. Unlike BMode, MMode has been designed specifically to assess diameter differences occurring within a single heartbeat. Therefore we believe that the detected decrease in circumferential strain by MMode is more likely to be correct, and that BMode should be avoided to assess circumferential strains.

Our data could not discern between long and short axis MMode for the assessment of circumferential strain, since both methods detected a highly significant difference between strain values at days 14 and 28. Nevertheless, both MMode methods also suffered from high standard deviation at all time points, even at baseline, and their mutual correlation was not convincing (Fig 6c and 6d). This can be explained in part by the large biological variability, as has been reported before. Another reason is that, even if the temporal resolution was sufficiently high to detect the exact moment of the foot and the peak of the wave, MMode was not sufficiently sensitive to detect the small differences in diameter corresponding to these peak time points (Fig 1a and 1b). This is related to the fact that the limited spatial resolution of regular MMode (typically around 30 μm) is not sufficient to detect the small differences in diameter (in this case 157 ± 35 μm at baseline and 116 ± 68 μm at days 14 and 28). We propose therefore that local stiffness should be assessed using RF data, in which the entire radio frequency spectrum (rather than its envelope) is used to reconstruct an image (see below).

### Global and regional PWV assessment with transit time methods

#### Control animals

In order to determine the effectiveness of transit time PWV assessed with non-invasive high-frequency ultrasound, we compared the method with invasive, pressure-based transit time PWV. In control animals, ultrasound-based PWV was not correlated to pressure-based PWV and showed a higher standard deviation (Fig 7). Two reasons can explain this result. In literature, as in the current paper, ultrasound-based PWV is usually assessed from Pulsed Doppler measurements. The foot of the velocity waveform is, however, less well defined and more difficult to detect than the foot of a pressure waveform due to the intrinsic variability and noise in the velocity estimators and the lower temporal resolution of the measurement [51, 52], inducing additional error in the denominator of the PWV equation. But, more importantly, ultrasound-based PWV is based on an external body tape measurement of the distance between ascending and distal abdominal measurement locations. This is similar to what happens in clinical practice in humans [53], but while the value of the measured distance is 10 times smaller in mice, the error on the tape measurement remains the same. This is not compensated for by a more accurate time lag measurement, because the increased temporal resolution (the higher probe frequency of 40 MHz and the shorter travel distance for the emitted ultrasound waves result in a round trip travel time that is 10 times higher than in a human setting) is needed to compensate for the increased heart rate (about 500 bpm in anesthetized mice, vs 60 bpm in humans). As a result, the combined error on aortic PWV in mice remains a factor 10 larger than in the clinical setting, which explains the observed high variation. Also, it limits the potential of using ultrasound to assess regional PWV in the abdominal region alone, since a halvation of the measured distance and transit time would result in an error increase by a factor 4. Despite its ubiquitous use in pre-clinical research [35, 41, 54] the use of ultrasound Pulsed Doppler to assess aortic PWV in mice thus has strong inherent shortcomings. Most of these shortcomings are corrected for when determining aortic PWV with a dual-sensor invasive pressure catheter. The distance between both measurement sensors is fixed (0.2 cm) so no distance measurement error is induced (Fig 3). The foot of the pressure wave is easily detected, and moreover both pressure waveforms are obtained simultaneously, so the obtained value cannot be influenced by a change in heart rate. Nevertheless, pressure-based transit time still resulted in a relatively high standard deviation in aortic PWV in control animals. One might hypothesize that the high standard deviation is due to the invasive nature of the experiment, disturbing local hemodynamics, or due to the longer exposure to anesthesia because of the surgery. However, while both of these factors may play a role in increasing the standard deviation, they are contradicted by the fact that the pressure-diameter method, in which local aortic stiffness is assessed without making use of transit times, the standard deviation is much lower in control animals. Our data therefore suggest that, in mice, stiffness assessment of longer segments of the aorta using the global transit time should be avoided and replaced by local stiffness assessment methods.

### Dissecting AAA animals

Neither ultrasound-based nor pressure-based transit time PWV could differentiate between controls and AAAs. Apart from the arguments mentioned above, introducing high standard deviations in both datasets, three important reasons explain this. First of all, in 50% of the animals, the specific dissecting AAA geometry prevented accurate transit time measurements, as the pressure sensor could not be moved far enough to allow for a measurement from the second sensor (Fig 3d). A second reason is that in case of aortic dissection the pulse wave travels over the inner tunica media, while the aortic stiffness that is measured with the local pressure-diameter method corresponds to the dissected tunica adventitia. The inner layer is, however, more distensible than the outer layer (Fig 2h), which contributes to lower PWV values obtained by transit time methods in dissecting AAAs. A third reason is that global and, to a lesser extent, regional transit time methods by definition also include healthy, non-diseased parts of the vessel which lowers the obtained stiffness value and makes it more difficult to discriminate between controls and dissecting AAAs.

### Local aortic stiffness assessment with simultaneous high-frequency ultrasound MMode and invasive pressure

Transit time methods, for various reasons described above, suffer from high standard deviations, (even in control animals) and moreover they could not discriminate between controls and AAAs. Local circumferential strains, assessed non-invasively with high-frequency ultrasound, could discriminate between baseline and different aneurysm time points in a large sample size, but also suffered from high standard deviations (even at baseline) and long and short axis methods did not correlate well. We have therefore performed a preliminary study to obtain local stiffness values in vivo from simultaneous recording of invasive pressure waveforms and long axis MMode diameter waveforms. The use of the long axis view for MMode diameter assessment seems in contradiction with earlier results that were in favour of short axis (Figs 4 and 5), but long axis was necessary in order to visualize the position of the pressure probe (Fig 3d). Unlike the MMode diameters processed to obtain systolic and diastolic diameters (Fig 1) however, in this case RF signals were obtained. We obtained very good morphological correspondance between diameter and pressure waveforms (Fig 3c), even in AAA mice, which demonstrates the reliability of diameter waveforms obtained by RF wall tracking. Moreover, the standard deviation in control animals was much lower than for transit time methods, and pressure-diameter assessment detected a significant difference in stiffness between controls and AAAs (Fig 7a). Also, this method only requires a single pressure sensor, and the sensor tip does not necessarily have to be inserted past the aneurysm. The effect of the pressure sensor on local hemodynamics in the aorta is thus greatly reduced. Therefore this method avoids many practical issues arising when assessing transit time invasively in dissecting AAAs. Our preliminary data therefore suggest that, if one is to perform invasive experiments to assess aortic stiffness in an accurate way, both in control and in angiotensin II-infused mice, this method is to be preferred over dual sensor transit time PWV measurements and other, non-invasive methods to assess local aortic stiffness such as circumferential strain. Multiple pressure-diameter measurements along the length of the aorta can be combined if a regional or global stiffness value is required.

## Conclusions

We have investigated different methods to assess aortic diameter, circumferential strain and aortic stiffness in vivo. Based on our observations, the following guidelines can be formulated:

- Aortic diameter should be assessed in short axis. When assessing aortic diameters in long axis MMode, one is likely to introduce an additional error due to the need to shift field of view to locate the maximal diameter. This is true for all mice, but the introduced error will be even larger in presence of dissecting AAA.
- Local methods to assess aortic stiffness are preferred over transit time methods that assess global PWV. This is true for all mice, and especially for mice with dissecting AAAs.
- When assessing global PWV using a transit time method, this should be done with an invasive dual-sensor pressure probe rather than with ultrasound transit time. This is true for all mice.
- If invasive measurements are feasible and accuracy is critical, local pressure-diameter measurements are to be preferred over non-invasive circumferential strains. This is true for all mice.
- When assessing non-invasive circumferential strain, this should be done with short or long axis MMode, and not with BMode. This is true for all mice, but the introduced error will be even larger in presence of dissecting AAA.
- In future studies, RF MMode might be an alternative to assess circumferential strain non-invasively as it provides accurate diameter waveforms rather than approximate peak values.
